# A prospective study on cancer risk after total hip replacements for 41,402 patients linked to the Cancer registry of Norway

**DOI:** 10.1186/s12891-020-03605-7

**Published:** 2020-09-08

**Authors:** Eva Dybvik, Ove Furnes, Leif I. Havelin, Sophie D. Fosså, Clement Trovik, Stein Atle Lie

**Affiliations:** 1 The Norwegian Arthroplasty Register, Department of Orthopaedic Surgery, Haukeland University Hospital, Bergen, Norway; 2 Department of Clinical Medicine, University of Bergen, Bergen, Norway; 3 National Resource Center for Late Effects after Cancer, Oslo University Hospital, the Norwegian Radium Hospital, Oslo, Norway; 4 Faculty of Medicine, University of Oslo, Oslo, Norway; 5 Musculoskeletal Tumour Centre, Department of Oncology, Haukeland University Hospital, Bergen, Norway; 6 Department of Clinical Science, University of Bergen, Bergen, Norway; 7 Department of Clinical Dentistry, University of Bergen, Bergen, Norway

**Keywords:** Total hip replacement, Cancer, Prosthesis fixation, Register study

## Abstract

**Background:**

Concerns have been raised that implants used in total hip replacements (THR) could lead to increased cancer risk. Several different materials, metals and fixation techniques are used in joint prostheses and different types of articulation can cause an increased invasion of particles or ions into the human body.

**Methods:**

Patients with THR registered in the Norwegian Arthroplasty Register during 1987–2009 were linked to the Cancer registry of Norway. Patients with THR due to osteoarthritis, under the age of 75 at time of surgery, were included. Standardized incidence ratios (SIR) were applied to compare cancer risk for THR patients to the general population. Types of THR were divided into cemented (both components), uncemented (both components), and hybrid (cemented femoral and uncemented acetabular components). To account for selection mechanisms, time dependent covariates were applied in Cox-regression, adjusting for cancer risk the first 10 years after surgery. The analyses were adjusted for age, gender and if the patient had additional THR-surgery in the same or the opposite hip. The study follows the STROBE guidelines.

**Results:**

Comparing patients with THR to the general population in Norway we found no differences in cancer risk. The overall SIR for the THR-patients after 10 years follow-up was 1.02 (95% CI: 0.97–1.07). For cemented THR, the SIR after 10 years follow-up was 0.99 (95% CI: 0.94–1.05), for uncemented, 1.16 (95% CI: 1.02–1.30), and for hybrid 1.12 (95% CI: 0.91–1.33). Adjusted Cox analyses showed that patients with uncemented THRs had an elevated risk for cancer (hazard ratio: HR = 1.24, 95% CI: 1.05–1.46, *p* = 0.009) when compared to patients with cemented THRs after 10 years follow-up. Stratified by gender the increased risk was only present for men. The risk for patients with hybrid THRs was not significantly increased (HR = 1.07, 95% CI: 0.85–1.35, *p* = 0.55) compared to patients with cemented THRs.

**Conclusions:**

THR patients had no increased risk for cancer compared to the general population. We found, however, that receiving an uncemented THR was associated with a small increased risk for cancer compared to cemented THR in males, but that this may be prone to unmeasured confounding.

## Background

In total hip replacement (THR) surgery, implants consisting of metals, polymers, and ceramics are inserted, some of which are fixated by means of bone cement. The numbers of metals and other materials, and the variety of sizes and bearing surfaces used in these implants over the years, have been substantial. Concerns have been raised whether the insertion of implants might lead to subsequent malignancies [1–4]. Tumours could hypothetically develop at the implant site, due to local reactions, or elsewhere in the body, caused by systemic influences. In animal studies, different materials have been used to model cancer development, but questions have been raised whether biomaterial-related tumours in animals have relevance to humans [5]. Most studies have found no increased cancer risk after THRs compared to the general population [6–16]. In a meta-analysis, Visuri and colleagues observed a decreased cancer risk for patients with arthroplasties [17]. Another meta-analysis did not confirm an overall increased cancer risk after THR and Total Knee Arthroplasty (TKA), but described an elevated risk for prostate cancer and melanomas [18]. A group from Sweden has reported an increased cancer risk among patients who had received a TKA due to osteoarthritis and rheumatoid arthritis, and also reported a latency effect for cancer after insertion of joint replacements [19].

In the present study, we have linked data on THRs from the Norwegian Arthroplasty Register (NAR) to the Cancer Registry of Norway (CRN). The primary aim of the study was to determine if there were differences in the long term (after 10 years) cancer risk according to types of prosthesis fixation; cemented (both femoral and acetabular components cemented), uncemented (both femoral and acetabular components uncemented), and hybrid (cemented femoral and uncemented acetabular component).

## Methods

In this prospective cohort study, follow-up time was measured from insertion of the initial prosthesis and until cancer, emigration, death, or December 31st 2009 (end of study), whichever came first.

The Norwegian Arthroplasty Register (NAR) started registration of total hip replacements (THR) in September 1987 [20]. More than 95% of patients receiving a THR are reported to the NAR [21, 22]. Patients registered with primary THR, from 1987 to 2009 with known prosthesis fixations, osteoarthritis, under the age of 75 at time of surgery, were followed from their first/initial operation. Since this study looks at the late (after 10 years) risk for cancer, patients older than 75 years were excluded. The selected patients were linked to the Cancer Registry of Norway (CRN) using the 11-digit personal identification number unique for all Norwegian citizens. The CRN was established in 1953 and registration of new cancer cases is compulsory. The registry has information on type of malignancy, date of diagnosis and initial treatment and demographics on 99% of all cancer patients in Norway [23, 24]. THR patients with cancer prior to the THR were excluded from the analysis. Hence, 41,402 patients were included. In the files from the CRN, ICD-7 code 189 (basal cell carcinoma) was not included.

Type of fixation was coded as fully cemented, fully uncemented, and hybrids (cemented femoral and uncemented acetabular components). THR patients with reversed hybrids (uncemented femoral and cemented acetabular components) were excluded due to few observations and short follow-up. The outcome variable of this study was the incidence of cancer occurring 10 years after insertion of THR. The Cancer Registry of Norway is a mandatory national health registry, regulated by law. All hospitals, laboratories, and general practitioners are obligated to report new cancer cases to the registry within 2 months. The Norwegian Arthroplasty Register (NAR), started in 1987, is a voluntary register licensed by the Data Inspectorate of Norway (16/01622–3). Patients give a written informed consent to be included in the registry. The operating surgeon reports the operation to the registry on a one-page standard form. The present study was approved by the Regional Committee for Medical and Health Research Ethics, Western Norway (170.06) and The Norwegian Data Protection Authority (06/01218–2). The study is reported according to the STROBE guidelines.

Standardized incidence ratio (SIR) was calculated to quantify the difference in cancer risk between THR patients and the population with corresponding age, gender and calendar year. The SIR will equal the hazard rate for patients divided with the hazard for the corresponding population [25, 26]. SIRs after 10 years follow-up were the main focus, but SIRs for the complete follow-up and SIRs before 10 years follow-up were also examined. SIR before 10 years follow-up may indicate possible selections of healthier or sicker patients for the different types of prosthesis. A SIR below 1 can indicate healthier patients than the population, while a SIR above 1 indicate sicker patients.

A Cox proportional hazards regression model, for the risk for cancer after 10 years follow-up, including a time-dependent indicator to adjust for the hazard for cancer in the first decade was set up to adjust for potential selection mechanisms. Hence, in this model, the hazard ratios after 10 years follow-up between the different types of prosthesis fixation were adjusted for the baseline hazards (cancer risk) the first 10 years follow-up. This difference-in-difference model minimizes the effect of unknown covariates. The regression models were adjusted for gender, age at operation, and time-dependent covariates for time to a contralateral THR and/or a revision operation. For testing of the proportionality assumption, a test of the Schoenfeld residuals after fitting the Cox-model was performed. To see if the results were consistent within subgroups, the analysis was stratified based on the age-categories and gender. Follow-up time was measured from insertion of the initial prosthesis and until cancer, emigration, death, or December 31st 2009 (end of study), whichever came first. Median follow-up was calculated using the inverse Kaplan-Meier method [27]. IBM-SPSS version 22 (IBM-SPSS, Chicago Ill), Stata (version 13-IC), and Fortran [26] were used for the statistical analyses. *P*-values less than 0.05 (5%) were considered statistically significant.

## Results

There were 13,954 (34%) males and 27,448 (66%) females, with a mean age of 65 (SD = 7) and 66 (SD = 7) respectively. Of the THR patients, 6167 were diagnosed with at least one cancer after THR, 1789 of these occurring more than 10 years after the first primary hip implant (Table 1). There was huge variation in the types of cancer and the most common single cancer type after 10 years follow-up was prostate cancer (257 males) and breast cancer (183 females) (Table 2). Total person years in the study were 453,950 and median follow-up was 11.9 years (Table 1). Uncemented THRs were predominantly given to younger and healthier individuals. For the included patients, the mean age for uncemented THR was 58.1 (sd = 8.3) years, for cemented 67.0 (sd = 5.9) years, while for hybrid prostheses mean age was 63.3 (sd = 7.2) years.

**Table 1:** Number of hips, cases of cancer, males and follow-up time

	Number of hips	Number of cancers	Percent males	Person years	Median follow-up	Hips after 10 years	Cancer after 10 years
**Total**							
**Cemented**	32,534	5060	32	361,924	12.2	16,653	1417
**Uncemented**	6679	743	41	65,634	9.8	2907	262
**Hybrid**	2189	364	36	26,392	13.3	1391	110
**All fixations**	41,402	6167	34	453,950	11.9	20,951	1789
**< 55 years**							
**Cemented**	1138	89	41	11,602	9.3	478	34
**Uncemented**	1967	139	46	22,174	11.9	1106	69
**Hybrid**	269	18	45	2705	10.6	144	6
**All fixations**	3374	246	44	36,481	10.8	1728	109
**55–64 years**							
**Cemented**	8480	1119	34	92,572	11.2	4167	391
**Uncemented**	3219	418	40	31,528	9.9	1398	151
**Hybrid**	801	172	36	9260	12.4	490	38
**All fixations**	12,500	1664	36	133,360	11.1	6055	580
**65–74 years**							
**Cemented**	22,916	3852	31	257,750	12.8	12,008	992
**Uncemented**	1493	186	38	11,932	7.2	403	42
**Hybrid**	1119	219	34	14,427	15.0	757	66
**All fixations**	25,528	4257	31	284,109	12.6	13,168	1100
**Men**							
**Cemented**	10,423	2037	100	112,322	12.2	5058	537
**Uncemented**	2748	343	100	26,690	9.8	1201	129
**Hybrid**	783	162	100	8993	12.8	472	44
**All fixations**	13,954	2542	100	148,005	11.9	6731	710
**Women**							
**Cemented**	22,111	3023	0	249,601	12.2	11,595	880
**Uncemented**	3931	400	0	38,944	9.8	1706	133
**Hybrid**	1406	202	0	17,399	13.7	919	65
**All fixations**	27,448	3625	0	305,945	12.0	14,220	1079

**Table 2:** Types of cancer following a total hip replacement, with more than 100 observed cases, before and after 10 years follow-up

	Before 10 years			After 10 years			Total
Cancer type (ICD-7)	Cemented	Uncemented	Hybrid	Cemented	Uncemented	Hybrid	
**Men**	1500	214	118	537	129	44	**2542**
**Prostate (177)**	547	66	37	191	51	15	**907**
**Large intestine (153)**	142	18	11	52	6	4	**233**
**Bronchus and trachea (162)**	128	20	12	43	13	3	**219**
**Skin (191)**	94	13	2	44	10	4	**167**
**Bladder and urinary organs (181)**	89	10	10	37	5	3	**154**
**Haematopoietic (207)**	72	11	7	27	8	0	**125**
**Malignant melanoma (190)**	64	10	7	17	2	4	**104**
**Rectum (154)**	64	11	4	16	5	1	**101**
**Others (*n*** **< 100)**	300	55	28	110	29	10	**532**
**Women**	2143	267	136	880	133	66	**3625**
**Breast (170)**	395	66	30	149	24	10	**674**
**Large intestine (153)**	328	32	24	131	15	9	**539**
**Bronchus and trachea (162)**	174	19	5	75	9	5	**287**
**Uteri (171 & 172)**	168	31	12	54	8	4	**277**
**Skin (191)**	116	5	2	74	5	8	**210**
**Rectum (154)**	103	15	6	31	9	2	**166**
**Haematopoietic (207)**	92	6	9	47	3	1	**158**
**Malignant melanoma (190)**	98	19	7	24	5	4	**157**
**Pancreas (157)**	74	8	3	41	11	3	**140**
**Ovary (175)**	92	7	3	28	3	2	**135**
**Brain and nervous system (193)**	66	11	5	29	8	2	**121**
**Kidney (180)**	76	8	5	24	2	1	**116**
**Lymphatic (206)**	53	7	11	26	10	4	**111**
**Bladder and urinary organs (181)**	61	6	3	25	5	0	**100**
**Other (n < 100)**	247	27	11	122	16	11	**434**
**Total**	3643	481	254	1417	262	110	**6167**

For THR-patients the overall standardized incidence ratio after 10 years follow-up was not statistically significantly different from the general population in Norway. SIR = 1.02, 95% CI: 0.97–1.07.

After 10 years follow-up, SIR for cemented prostheses was 0.99 (95% CI: 0.94–1.05), for uncemented, 1.16 (95% CI: 1.02–1.30) and for hybrid 1.12 (95% CI: 0.91 1.33) (Table 3). In the regression model we found that patients with uncemented prostheses, had an increased risk for cancer after 10 years follow-up compared to cemented prostheses (HR = 1.24, 95% CI: 1.05–1.46, *p* = 0.009). Patients with hybrid prostheses were not statistically significant from those with cemented prostheses (HR = 1.07, 95% CI: 0.85–1.35, *p* = 0.55). Both SIR and the Cox model gave statistically significant increased risk for cancer 10 years after receiving an uncemented total hip replacement (THR).

**Table 3:** SIR and Cox model with time dependent covariates for the excess risk after 10 years follow-up comparing different types of fixations

	#hips	#cancers	Total	Before 10 years	After 10 years	Cox model with time-dep. covariates^**a**^		
			SIR (95% CI)	SIR (95% CI)	SIR (95% CI)	HR ^**b**^	(95% CI)	P
**Total:**	41,402	6167	1.06 (1.03–1.09)	1.06 (1.03–1.09)	1.02 (0.97–1.07)			
**Cemented**	32,534	5060	1.05 (1.03–1.08)	1.06 (1.03–1.09)	0.99 (0.94–1.05)	1	(reference)	–
**Uncemented**	6679	743	1.08 (1.00–1.16)	1.04 (0.94–1.13)	1.16 (1.02–1.30)	**1.24**	(1.05–1.46)	**0.009**
**Hybrid**	2189	364	1.11 (0.99–1.22)	1.09 (0.95–1.22)	1.12 (0.91–1.33)	1.07	(0.85–1.35)	0.55
**< 55 years:**	3374	246	0.96 (0.84–1.08)	0.89 (0.74–1.04)	1.06 (0.86–1.26)			
**Cemented**	1138	89	1.09 (0.86–1.31)	1.04 (0.77–1.32)	1.16 (0.77–1.56)	1	(reference)	–
**Uncemented**	1967	139	0.89 (0.75–1.04)	0.80 (0.62–0.99)	1.00 (0.76–1.24)	1.19	(0.69–2.05)	0.54
**Hybrid**	269	18	0.96 (0.52–1.40)	0.85 (0.37–1.34)	1.27 (0.25–2.29)	1.31	(0.45–3.82)	0.62
**55–64 years:**	12,500	1664	1.06 (1.01–1.11)	1.00 (0.95–1.06)	1.16 (1.06–1.25)			
**Cemented**	8480	1119	1.02 (0.96–1.08)	0.97 (0.90–1.04)	1.11 (1.00–1.22)	1	(reference)	–
**Uncemented**	3219	418	1.15 (1.04–1.27)	1.08 (0.95–1.21)	1.29 (1.09–1.50)	1.07	(0.85–1.36)	0.56
**Hybrid**	801	127	1.15 (0.95–1.35)	1.13 (0.90–1.36)	1.16 (0.79–1.53)	0.93	(0.62–1.38)	0.71
**65–74 years:**	25,528	4257	1.07 (1.03–1.10)	1.09 (1.05–1.12)	0.96 (0.90–1.02)			
**Cemented**	22,916	3852	1.06 (1.03–1.10)	1.09 (1.05–1.13)	0.95 (0.89–1.01)	1	(reference)	–
**Uncemented**	1493	186	1.10 (0.94–1.26)	1.11 (0.93–1.29)	1.03 (0.72–1.35)	1.00	(0.71–1.43)	0.98
**Hybrid**	1119	219	1.10 (0.96–1.25)	1.09 (0.91–1.26)	1.09 (0.82–1.35)	1.12	(0.83–1.51)	0.46
**Men:**	13,954	2542	1.03 (0.99–1.07)	1.02 (0.97–1.07)	1,03 (0.95–1.10)			
**Cemented**	10,423	2037	1.02 (0.98–1.07)	1.02 (0.97–1.07)	0,99 (0.91–1.08)	1	(reference)	–
**Uncemented**	2748	343	1.04 (0.93–1.15)	0.98 (0.85–1.11)	1.15 (0.95–1.34)	**1.41**	**(1.11–1.80)**	**0.004**
**Hybrid**	783	162	1.13 (0.96–1.31)	1.11 (0.91–1.31)	1.15 (0.81–1.49)	1.11	(0.78–1.58)	0.58
**Women:**	27,448	3625	1.08 (1.05–1.12)	1.09 (1.05–1.13)	1.02 (0.96–1.08)			
**Cemented**	22,111	3023	1.08 (1.04–1.12)	1.09 (1.04–1.14)	1.00 (0.93–1.06)	1	(reference)	–
**Uncemented**	3931	400	1.12 (1.01–1.23)	1.09 (0.96–1.22)	1.17 (0.97–1.36)	1.09	(0.87–1.36)	0.47
**Hybrid**	1406	202	1.09 (0.94–1.24)	1.07 (0.89–1.24)	1.10 (0.80–1.39)	1.06	(0.78–1.43)	0.72

^a^ Adjusted for current age, gender, diagnosis, and a second primary or revision prosthesis operation

^b,c^ The estimates are hazard ratios (HR)

Testing of the proportionality assumption in the regression model showed that in a model with no time-dependent covariates, the dummy variable for uncemented (versus cemented) implants interacted with time (*p* = 0.008). However, for the fully adjusted model, with all the mentioned time-dependent covariates, none of the covariates had an interaction with time (overall *p* = 0.96).

Stratified by age categories, there were no significant differences in risk for cancer between the different types of fixation (Table 3). Males with uncemented THRs had an increased risk for cancer compared to males with cemented THRs (HR = 1.41, 95% CI: 1.11–1.80, *p* = 0.004), while this was not found for females (HR = 1.09, 95% CI: 0.87–1.36, *p* = 0.47). Stratified by gender and age categories we found no statistically significant differences comparing uncemented and hybrid prostheses to cemented prostheses (Table 4).

**Table 4:** Cox model with time dependent covariates for subgroups of gender and age groups

	# hips	# cancer	Total	Before 10 years	After 10 years	Cox model with timedep covariates		
			SIR (95% CI)	SIR (95% CI)	SIR (95% CI)	HR	(95% CI)	P
**Men**								
< 55 years								
Cemented	471	35	1.16 (0.78–1.55)	1.11 (0.63–1.58)	1.23 (0.59–1.88)	1	(reference)	
Uncemented	896	68	0.98 (0.75–1.21)	1.00 (0.67–1.33)	0.94 (0.62–1.26)	0.91	(0.39–2.08)	0.82
Hybrid	121	7	0.87 (0.23–1.52)	0.68 (0.01–1.35)	1.37 (0.00–2.92)	1.66	(0.32–8.63)	0.55
55–64 years								
Cemented	2870	463	0.99 (0.90–1.08)	0.93 (0.82–1.03)	1.10 (0.93–1.26)	1	(reference)	
Uncemented	1281	184	1.03 (0.88–1.18)	0.91 (0.74–1.08)	1.26 (0.97–1.54)	1.26	(0.89–1.79)	0.19
Hybrid	287	52	1.05 (0.77–1.34)	1.03 (0.69–1.36)	1.09 (0.56–1.62)	0.94	(0.50–1.74)	0.83
65–74 years								
Cemented	7082	1539	1.03 (0.98–1.08)	1.04 (0.98–1.10)	0.94 (0.84–1.04)	1	(reference)	
Uncemented	571	91	1.12 (0.89–1.35)	1.09 (0.84–1.35)	1.18 (0.65–1.71)	1.14	(0.68–1.90)	0.63
Hybrid	375	103	1.20 (0.97–1.43)	1.20 (0.93–1.46)	1.17 (0.71–1.63)	1.09	(0.69–1.72)	0.70
**Women**								
< 55 years								
Cemented	667	54	1.04 (0.77–1.32)	1.00 (0.67–1.34)	1.12 (0.63–1.61)	1	(reference)	
Uncemented	1071	71	0.83 (0.63–1.02)	0.67 (0.45–0.89)	1.06 (0.72–1.41)	1.44	(0.70–2.98)	0.32
Hybrid	148	11	1.03 (0.42–1.63)	0.98 (0.30–1.66)	1.18 (0.00–2.52)	1.07	(0.25–4.50)	0.93
55–64 years								
Cemented	5610	656	1.04 (0.96–1.12)	1.00 (0.90–1.09)	1.12 (0.98–1.27)	1	(reference)	
Uncemented	1938	234	1.27 (1.11–1.43)	1.24 (1.04–1.43)	1.33 (1.30–1.36)	0.94	(0.68–1.29)	0.69
Hybrid	514	75	1,22 (0,95-1,50)	1,21 (0,89-1,54)	1,21 (0,71-1,72)	0.91	(0.54–1.54)	0.73
65–74 years								
Cemented	15,834	2313	1.09 (1.04–1.13)	1.12 (1.06–1.17)	0.95 (0.88–1.03)	1	(reference)	
Uncemented	922	95	1.08 (0.86–1.3)	1.12 (0.86–1.38)	0.93 (0.54–1.32)	0.90	(0.56–1.44)	0.65
Hybrid	744	116	1.03 (0.84–1.21)	0.99 (0.77–1.21)	1.04 (0.72–1.36)	1.14	(0.78–1.68)	0.50

## Discussion

There has been focus on the risk for cancer after insertion of joint replacements [2–4]. The majority of studies are from the national arthroplasty registries in Finland [9, 10, 12, 15, 16], Scotland [6], England and Wales [14], and Sweden [19]. All but one of these studies conclude that there is no (or a negligible) increased risk for cancer after insertion of joint replacements. On the other hand, Wagner and colleagues have reported an overall increased cancer risk for total knee arthroplasty (TKA) patients compared to the general population. In addition, they reported findings of specific cancer types, which they argue can be a result of TKA exposure [19]. Our study supports previous findings showing no overall increased risk for cancer after THR, neither before nor after 10 years follow-up. For uncemented THRs we found an association with a small increased risk for cancer for males.

There are limitations in this study. As shown by Lie and colleagues [28], patients with a THR have reduced overall mortality compared to the general population, while THR patients under 60 years have increased mortality and patients over 80 years of age have considerably reduced mortality compared to the population in general. Furthermore, uncemented prostheses have predominantly been given to younger and healthier patients, while cemented prostheses have been given to elderly and frailer patients [29]. Consequently, adjusting for the risk for cancer, the first 10 years after primary THR (difference-in-difference model) would adjust for (unknown) risk factors contributing to the baseline risk for cancer for the different categories of patients. Still, there is a potential for a complex selection mechanism for receiving a THR and also for receiving the different types of THR, which we are not able to adjust for. Hypothetically, receiving a THR can increase the attention to own health. Subsequently, this can lead to more visits for medical care (e.g. general practitioner), which may increase the number of tests and also the probability of being tested for cancer. This would particularly be the case for prostate cancer in men.

Recently, Cartilage Oligomeric Matrix Protein (COMP), which plays an important role in the organization of the extracellular matrix of cartilage, has been identified as a potent driver of the progression of prostate cancer, acting in an anti-apoptotic fashion by interfering with the Ca2+ homeostasis of cancer cells [30]. In a retrospective case control series in prostate cancer patients with and without osteoarthritis, this condition was identified as an independent risk factor for metastatic disease. However, when joint arthroplasty was included in the model, osteoarthritis was no longer an independent risk factor [31]. It is unlikely that this association can explain the small increase in cancer risk in men with uncemented compared to cemented THR in our study.

The common analytical approach to study cancer risk for THR and TKA patients is to compare the observed cancer risk for arthroplasty patients with cancer rates in the general population. When SIRs are used to compare the cancer risk for the patients studied with the cancer risk in the population, it is assumed that prosthesis patients are comparable to the general population. Previous studies find no increase in risk for cancer after an arthroplasty compared to the general population [6–18]. Overall, this agrees with our finding, using the same analyses techniques.

From studies of secondary cancer related to anti-neoplastic treatment, it is known that the latency from the first to the subsequent malignant tumour is 10 years or more [32, 33]. In the regression models in this study, we took into consideration that the development of cancer related to arthroplasty can take at least one decade, and that cancer diagnoses during the first years after a THR operation are most likely related to factors other than the arthroplasty itself. To compare the different types of fixations, we therefore used baseline cancer risk at the first 10 years follow-up as a reference. In these analyses we thus compared the difference in cancer risk between different types of fixations, and other factors, adjusting for the crucial selection for receiving the THR.

We found an increased cancer risk for patients with two uncemented prostheses components, compared to patients where both prostheses components were cemented. Patient with hybrid prostheses had not a statistically increased risk for cancer compared to patients with two cemented components.

In analyses of cancer after THR, death is a competing risk. For the present analyses we did not take competing risks into account. The reason is that since there are differences in selection mechanisms between the different prosthesis, which will be apparent in analyses with death as endpoint, death may also be a collider in causal terminology when we study the risk for receiving cancer. Accordingly, using models for competing risks, a false and elevated risk between the types of THR and cancer was present (analyses not shown). The relative differences in the SIRs in our analyses correspond to the findings from the Cox model with time-dependent adjustment, which we consider strengthen our findings.

There has been a concern about cancer risks associated with metal on metal articulations for THRs [9, 10, 14, 34], but other and newer types of articulations should also be studied [35]. Articulation has not been included in the present study since the majority of THR prostheses in the Norwegian Arthroplasty Register have a metal head and polyethylene cup, and other articulations have lower numbers or shorter follow-up [22]. Metal-on-metal has rarely been used in Norway in the study period. Only approximately 200 cemented and less than 200 uncemented implants of this type, most of which with small heads (< 32 mm) were used in the time period studied [36]. Metal on metal resurfacing implants were excluded from the study, because this type of THR is a marginal problem in this study, and omitting these implants would not alter our findings.

## Conclusion

In the present study, we found no increased risk for cancer in THR patients compared to the general population. However, we found a small increased risk for cancer after insertion of THR where both components were uncemented, compared to prostheses where both were cemented. In gender-stratified analysis, this increased risk was only found for men, but not found in age-stratified analysis for men. The difference was small and prone to unmeasured confounding. The risk for cancer after joint replacements and possible mechanisms related to cancer for patients with musculoskeletal diseases and/or joint replacements should be studied further. Surveillance of new products, materials and prostheses, with respect to rare and adverse outcomes like cancer, is important, also in the future.

## Data Availability

The datasets in this study are based on two national Norwegian databases. Linkage of the databases was achieved as described in the “Ethics approval and consent to participate” section of the article. Access of the dataset was restricted to the authors of the article. Further details of the data can be obtained by contacting the corresponding author.

## Abbreviations

CI: Confidence Interval
CRN: Cancer Registry of Norway
HR: Hazard Ratio
NAR: Norwegian Arthroplasty Register
SD: Standard Deviation
SIR: Standardized Incidence Ratio
STROBE: Strengthening the Reporting of Observational studies in Epidemiology
THR: Total Hip Replacement
TKA: Total Knee Arthroplasty
